# TIGER: A Web Portal of Tumor Immunotherapy Gene Expression Resource

**DOI:** 10.1016/j.gpb.2022.08.004

**Published:** 2022-08-29

**Authors:** Zhihang Chen, Ziwei Luo, Di Zhang, Huiqin Li, Xuefei Liu, Kaiyu Zhu, Hongwan Zhang, Zongping Wang, Penghui Zhou, Jian Ren, An Zhao, Zhixiang Zuo

**Affiliations:** 1 State Key Laboratory of Oncology in South China, Cancer Center, Collaborative Innovation Center for Cancer Medicine, School of Life Sciences, Sun Yat-sen University, Guangzhou 510060, China; 2 Department of Gastrointestinal Surgery, Guangdong Provincial Key Laboratory of Colorectal and Pelvic Floor Diseases, Guangdong Institute of Gastroenterology, The Sixth Affiliated Hospital, Sun Yat-sen University, Guangzhou 510655, China; 3 Department of Urology, Cancer Hospital of the University of Chinese Academy of Sciences (Zhejiang Cancer Hospital), Hangzhou 310016, China; 4 Experimental Research Center, Cancer Hospital of the University of Chinese Academy of Sciences (Zhejiang Cancer Hospital), Hangzhou 310016, China; 5 Institute of Cancer and Basic Medicine (IBMC), Chinese Academy of Sciences, Hangzhou 310000, China

**Keywords:** Immunotherapy, Biomarker, Gene expression, Single-cell RNA-seq, Web server

## Abstract

**Immunotherapy** is a promising cancer treatment method; however, only a few patients benefit from it. The development of new immunotherapy strategies and effective **biomarkers** of response and resistance is urgently needed. Recently, high-throughput bulk and single-cell **gene expression** profiling technologies have generated valuable resources. However, these resources are not well organized and systematic analysis is difficult. Here, we present TIGER, a tumor immunotherapy gene expression resource, which contains bulk transcriptome data of 1508 tumor samples with clinical immunotherapy outcomes and 11,057 tumor/normal samples without clinical immunotherapy outcomes, as well as single-cell transcriptome data of 2,116,945 immune cells from 655 samples. TIGER provides many useful modules for analyzing collected and user-provided data. Using the resource in TIGER, we identified a tumor-enriched subset of CD4^+^ T cells. Patients with melanoma with a higher signature score of this subset have a significantly better response and survival under immunotherapy. We believe that TIGER will be helpful in understanding anti-tumor immunity mechanisms and discovering effective biomarkers. TIGER is freely accessible at http://tiger.canceromics.org/.

## Introduction

Immunotherapy is a promising cancer treatment method that utilizes the immune defense system against cancer. Among the different types of immunotherapy techniques, immune checkpoint blockade (ICB) has revolutionized the treatment of advanced cancers. ICB has shown durable responses in patients with various cancer types [1], [2]; however, most patients with cancer cannot benefit from ICB because of the low response rates in many cancer types. Although considerable progress has been achieved, efforts are still needed to explore new immunotherapy methods and discover effective biomarkers of response and resistance.

Gene expression data have shown broad applications in identifying biomarkers to predict drug responses in cancer treatment [3], [4]. In recent years, advances in high-throughput technologies have generated large amounts of transcriptomic gene expression data from cancer samples, providing valuable resources for research related to cancer immunotherapy. Some cancer projects, such as The Cancer Genome Atlas (TCGA) [5], have generated transcriptomic gene expression data for thousands of tumor samples without the use of immunotherapy information across multiple cancer types. Although these transcriptomic gene expression data were not originally designed to study cancer immunotherapy, recent studies have reported that analyses of these data could improve our understanding of tumor–immune cell interactions, thus facilitating the identification of cancer immunotherapy response biomarkers [6], [7]. More recently, the amount of transcriptomic gene expression data with clinical information of cancer immunotherapy has grown rapidly, which enables the use of gene expression signatures to predict immunotherapy responses [8], [9]. Despite these efforts, the effectiveness of immunotherapy response biomarkers remains an open question because of the small sample size of each dataset. Recent explosively growing single-cell transcriptome studies have provided a better understanding of immune cell profiles in the tumor microenvironment (TME) before and after immunotherapy at a single-cell resolution [10], [11], [12], [13], [14], [15], [16], [17]. We hypothesized that integrative analysis of large-scale public bulk and single-cell cancer transcriptome data would be helpful for comprehensively exploring tumor–immune cell interactions and developing reliable immunotherapy response prediction biomarkers.

Currently, several web servers have been developed for the analysis of gene expression resources related to cancer immunotherapy. Tools such as CIBERSORT [18], the Cancer Imaging Archive (TCIA) [7], Tumor Immune Estimation Resource (TIMER) [19], and ImmuCellAI [20] provide useful functions for mining the immune cell infiltration in solid cancers based on TCGA or user-provided bulk gene expression data. Tumor Immune Dysfunction and Exclusion (TIDE) [21] and TISIDB [22] allow users to comprehensively evaluate biomarkers of immunotherapy response and resistance based on public bulk gene expression datasets with or without clinical immunotherapy information. Tumor Immune Single Cell Hub (TISCH) (https://tisch.comp-genomics.org) [23] and Single Cell Portal (https://singlecell.broadinstitute.org) provide interfaces for visualizing and analyzing the public single-cell RNA sequencing (scRNA-seq) datasets of human tumors. Although these tools are very useful in exploring cancer immunology, an integrative resource of cancer bulk and single-cell transcriptome data specialized for cancer immunotherapy research is still lacking. In addition, although Single Cell Portal and TISCH have implemented single-cell analysis methods such as clustering analysis, differential gene expression analysis, and cell type annotation, it lacks many additional frequently used single-cell analysis functions. For example, differential analysis between tumor and normal, gene co-expression analysis, trajectory analysis, and cell–cell communication analysis are important for understanding anti-tumor immunity, but these functions are not available in Single Cell Portal and TISCH. Therefore, we developed Tumor Immunotherapy Gene Expression Resource (TIGER; http://tiger.canceromics.org/), a web-accessible portal for the integrative analysis of bulk and single-cell cancer transcriptome data, which is dedicated to facilitating the development of new immunotherapy methods and effective biomarkers.

## Web server content and methods

### Data sources

Preprocessed TCGA bulk RNA sequencing (RNA-seq) data of tumor and normal samples were downloaded from the website of the UCSC Xena project (https://xena.ucsc.edu). The bulk RNA-seq and gene expression microarray data of tumor samples with clinical immunotherapy information were collected from the Gene Expression Omnibus (GEO; https://ncbi.nlm.nih.gov/geo) and Sequence Read Archive (SRA; https://ncbi.nlm.nih.gov/sra) databases by searching for keywords such as immunotherapy, programmed cell death protein 1 (*PD-1*) inhibitors, and cytotoxic T-lymphocyte antigen 4 (*CLTA4*) inhibitors. Preprocessed data were used if raw data were not available. scRNA-seq data of human tumors were collected from the GEO, Genome Sequence Archive (GSA; https://ngdc.cncb.ac.cn/gsa-human/), European Molecular Biology Laboratory’s European Bioinformatics Institute (EMBL-EBI; https://www.ebi.ac.uk), and Single Cell Portal databases by searching for keywords such as single-cell, scRNA-seq, 10x Genomics, inDrop, and Smart-seq2. The clustered regularly interspaced short palindromic repeats (CRISPR) data related to tumor immunology were collected from the GEO and SRA databases.

### Analysis of scRNA-seq data

#### Data preprocessing

STAR was used to align the FASTQ format reads to the human reference genome (hg38 and GRCh38) [24], and then Cell Ranger was used to export the gene expression matrix. Quality control was performed for each scRNA-seq dataset using the procedure implemented in Seurat (v3.1.3) R toolkit [25]. First, cells with more than 10% mitochondrial RNA content were considered dead or dying and were removed. Cells with feature counts less than 200 or more than 3000 were also excluded. Cells expressing more than one of these three markers (*CD2*, *CD79A*, and *CD68*) simultaneously were defined as doublets and removed. Secondly, the filtered gene expression matrix for each sample was normalized by the “NormalizeData” function of Seurat, and the highly variable genes were retained by the “FindVariableFeatures” function. Lastly, “FindIntegrationAnchors” and “Integratedata” functions were used to integrate the gene expression matrices of all samples, in which batch effects between different samples were adjusted.

#### Single-cell clustering and cell type annotation

Seurat (v3.1.3) was used to cluster the cells based on single-cell expression profiles. First, “RunPCA” function in Seurat was used to perform the principal component analysis (PCA), and “FindNeighbors” function in Seurat was used to construct a K-nearest neighbor graph. Next, the most representative principal components (PCs) selected based on PCA were used for clustering analysis with “FindCluster” function in Seurat. Finally, a Uniform Manifold Approximation and Projection (UMAP) algorithm [26] was used to visualize the different clusters.

We then used classical cell markers to annotate the cell types. According to the results of differential expression analysis among cell types, cells with significantly up-regulated genes such as *CD2*, *CD3D*, and *CD3E* were annotated as T cells, cells with significantly up-regulated genes such as *CD79A*, *CD19*, and *MS4A1* were annotated as B cells, cells with significantly up-regulated genes such as *IGHA1*, *TNFRSF17*, and *SDC1* were annotated as plasma cells, cells with significantly up-regulated genes such as *CD14*, *FCGR3A*, and *CD68* were annotated as myeloid cells, cells with significantly up-regulated genes such as *VWF*, *CDH5*, and *FLT1* were annotated as endothelial cells, cells with significantly up-regulated genes such as *DCN*, *COL1A1*, and *ACTA2* were annotated as fibroblast cells, cells with significantly up-regulated genes such as *KRT18*, *KRT8*, and *EPCAM* were annotated as malignant/epithelial cells, cells with significantly up-regulated genes such as *MS4A2*, *CPA3*, and *TPSB2* were annotated as mast cells, and cells with significantly up-regulated genes such as *NCAM1*, *KLRB1*, and *NCR3* were annotated as natural killer (NK)/natural killer T (NKT) cells. *CD4* and *CD8* gene expression levels were used to differentiate between CD4^+^ and CD8^+^ T cells. To get higher resolution clusters in CD4^+^ T cell, CD8^+^ T cell, B cell, and myeloid cell, the “resolution” parameter used in “FindCluster” was set from 0.5 to 0.8.

#### Differential expression analysis

The differential expression analysis for deriving cell type markers and differentially expressed genes between different sample groups such as tumor and normal was performed with “wilcoxauc” function in Presto [27].

#### Pathway/gene set analysis

Pathway/gene set enrichment analysis was performed using the Correlation Adjusted MEan RAnk gene set test (CAMERA) [28], which was implemented in the singleseqgset (version 0.1.2) R package. In brief, the log_2_ fold change in the mean expression level of a specific gene between the specific cell cluster and other cells was used as the test statistic. The 50 hallmark gene sets in the MSigDB database (https://www.gsea-msigdb.org/gsea/msigdb) were used for the CAMERA analysis.

#### Correlation analysis

Spearman’s rank or Pearson’s correlation coefficient was used to evaluate the correlation between different gene pairs in a specific cell type.

#### Trajectory analysis

Monocle 2 [29] was used to reconstruct the single-cell trajectories. Briefly, the “negbinomial.size” function was used to create a “CellDataSet” object from the unique molecular identifier (UMI) count matrices with default settings. The variable genes were defined using the following cutoff: dispersion_empirical > dispersion_fit, and mean expression > 0.001. Dimensional reduction was performed using the “DDRTree” method, and cell ordering was performed using the “orderCells” function.

#### Cell–cell communication analysis

CellPhoneDB (version 2.0.6) was used for ligand–receptor analysis to investigate potential cell–cell communication between different cell types [30]. The algorithm used by CellPhoneDB only considers receptors and ligands that are highly expressed in the test cell type and then calculates the cell type-specific likelihood of a given receptor–ligand complex with a sufficient number of arrangements. In addition, we permuted the change in cell type label for each cell 1000 times to calculate the significance of each pair. The *P* value of the cell–cell communication was calculated using the ratio of the mean for a particular receptor–ligand pair to the mean distribution for a random arrangement.

### Analysis of bulk gene expression data

#### Data preprocessing

TCGA raw data were preprocessed using the UCSC Xena project. The bulk RNA-seq raw data from other sources were preprocessed using FastQC to check the quality of the sequencing reads. Samples with low sequencing quality were removed. Sequencing reads were processed using Cutadapt [31] to remove adapters and low-quality end bases. The processed reads were aligned to human reference genomes (hg38 and GRCh38) using STAR [24]. featureCounts [32] was used to derive the read counts for each gene, which were normalized to fragments per kilobase of transcript per million (FPKM) values.

#### Differential expression analysis

For the bulk RNA-seq and gene expression microarray data, differential expression analysis was performed using the Wilcoxon rank-sum test. The value of the interactive heatmap in the differential expression analysis of the immunotherapy response module was calculated using the following formula: $-SIGN\left(log2\left(FC\right)\right)\times log10\left(P\right)$, where FC represents the fold change and P represents the *P* value derived from the Wilcoxon rank-sum test.

#### Survival analysis

The association between gene expression and overall survival in the immunotherapy data was calculated using univariate Cox regression analysis. The value of the interactive heatmap in the survival analysis of the immunotherapy response module was calculated using the following formula: $-SIGN\left(log2\left(HR\right)\right)\times log10\left(P\right)$, where HR represents the hazard ratio and P represents the *P* value derived from univariate Cox regression analysis.

#### Correlation analysis

The correlation between the expression of gene–gene pairs was calculated using Spearman’s rank or Pearson’s correlation coefficient.

#### Gene set score

The average expression of all the genes in the gene set represents the gene set score.

#### Prediction of immunotherapy response

The gene signatures for predicting immunotherapy responses were obtained from the literature. The score of each gene signature was calculated according to the parameters used in the original study. We applied a robust rank aggregation algorithm [33] to integrate all gene signature scores in an unbiased manner. The aggregation rank score was used to predict the immunotherapy response in patients with cancer.

### Web server implementations

All data in TIGER were stored and managed in MySQL tables, JSON files, Rds, and RData files. Web interfaces were implemented using PHP, HTML, JavaScript, and CSS. Statistical diagrams were generated using ECharts and Rscripts.

## Results

### Data summary

TIGER contains bulk transcriptome gene expression data of 1508 tumor samples of 8 cancer types with clinical immunotherapy information from 20 published studies and 11,057 tumor/normal samples of 33 cancer types without the clinical immunotherapy information of TCGA. Moreover, TIGER contains scRNA-seq data of 2,116,945 immune cells from 655 samples of 25 cancer types, including clinical immunotherapy information of 119,039 immune cells from 63 samples. In addition, we collected 31 CRISPR screening datasets from studies that identified genes responsible for the anti-tumor immune response.

### Web interface and usage

**Figure 1:**
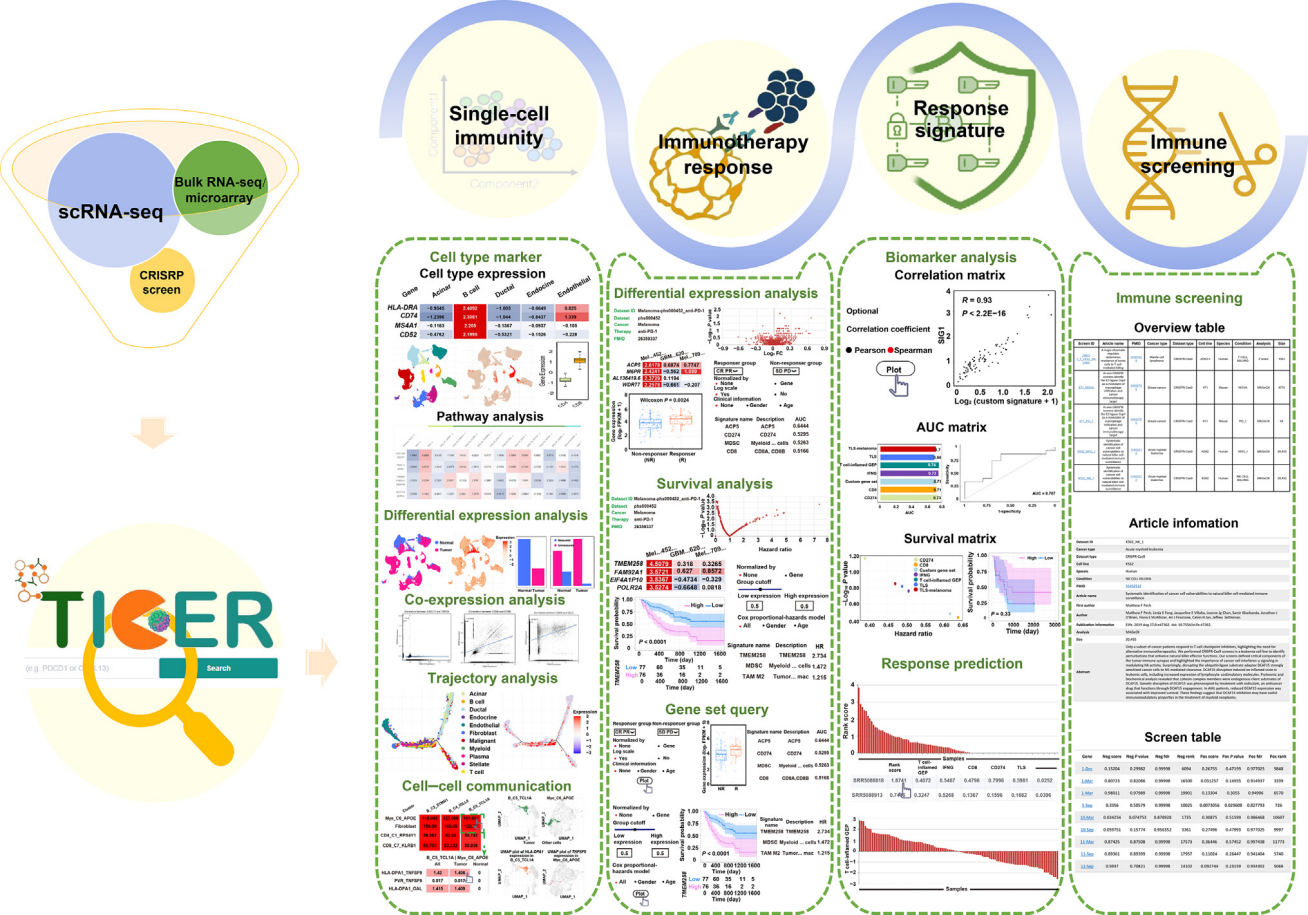
**Overall design and construction of TIGER** TIGER aims to help researchers unearth potential mechanisms in anti-tumor immunity and discover potential biomarkers by integrating tumor immunology-related bulk transcriptome, single-cell transcriptome, and immune screening data. TIGER provides four functional analysis modules, including single-cell immunity, immunotherapy response, response signature, and immune screening, to allow users to access the resources in TIGER. TIGER, tumor immunotherapy gene expression resource; RNA-seq, RNA sequencing; scRNA-seq, single-cell RNA sequencing; AUC, area under the curve; FC, fold change; FPKM, fragments per kilobase of transcript per million.

TIGER integrates the collected data into four modules: single-cell immunity, immunotherapy response, response signature, and immune screening. It provides user-friendly web interfaces to access the four modules (Figure 1; Video S1).

#### Single-cell immunity

**Figure 2:**
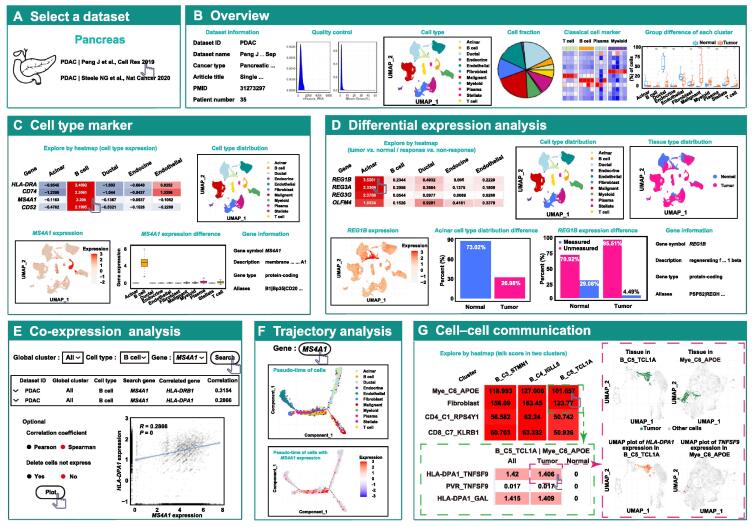
**Web interface and usage of “single-cell immunity” module in TIGER** **A.** The interface for users to select the ones that they are interested in. **B.** Overview tab. First in the first row: dataset information. Second in the first row: density plot showing quality control. Third in the first row: UMAP plot showing the pre-analyzed clustering results of selected main lineage cells. Fourth in the first row: pie plot showing the proportion of each of main lineage cells. Fifth in the first row: heatmap showing the classical cell markers of each of the main lineage cells. Sixth in the first row: boxplot comparing the percentage of cell number of the main lineage cells between normal and tumor. **C.** Cell type marker tab. First in the first row: heatmap showing the expression of gene markers in each cell type. Second in the first row: UMAP plot showing the cell types of the main lineage cells (color-coded for cell types). First in the second row: UMAP plot showing the expression of the selected gene in the selected main lineage cells (color-coded for gene expression abundance). Second in the second row: boxplot showing the differential expression of the selected gene in different cell types of the selected main lineage cells. Third in the second row: gene information. **D.** Differential expression analysis tab. First in the first row: heatmap showing the differential expression of all genes in each cell type between tumor and normal or between response and non-response. Second in the first row: UMAP plot showing the cell types of the selected main lineage cells (color-coded for cell types). Third in the first row: UMAP plot showing the tissue types of the selected main lineage cells (color-coded for tissue types). First in the second row: UMAP plot showing the expression of the selected gene in the selected main lineage cells (color-coded for gene expression abundance). Second in the second row: barplot showing the selected cell type distribution difference between tumor and normal. Third in the second row: barplot showing the differential expression of the selected gene between tumor and normal. Fourth in the second row: gene information. **E.** Co-expression analysis tab. Top: table of the correlation of the selected gene and relevant gene in selected dataset. Bottom: a plot visualizing the correlation of the selected gene and relevant gene. **F.** Trajectory analysis tab. The trajectory analysis showing the expression of the genes of interest in the pseudotemporal ordering of cells. **G.** Cell–cell communication tab. UMAP, uniform manifold approximation and projection.

This module provides users with plentiful scRNA-seq data analysis functions in six tabs, including “overview”, “cell type marker”, “differential expression analysis”, “co-expression analysis”, “trajectory analysis”, and “cell–cell communication” tabs. We provided a total of 40 datasets of 18 cancer types for user selection (Figure 2A). First, users can obtain basic information regarding the selected dataset and number of immune cells of each type in the dataset (Figure 2B). Users can obtain the dataset source, cell number, cell type information, and quality control density graph in the “overview” tab. In the “cell type marker” tab, an interactive heatmap in a sub-tab showing the fold changes in gene expression between a cell cluster and other cells is presented to allow users to explore the markers and functions of each cell type (Figure 2C). In the interactive heatmap, a selection box is provided to allow users to quickly locate the main lineage cell types of interest, and a text box is provided to allow users to search for genes of interest (Figure 2C). Moreover, users can sort genes based on fold changes in the cell type of interest. By clicking on a cell in the interactive heatmap, users will obtain detailed information on the expression of the selected gene in the selected cell type. For detailed information, UMAP plots and a boxplot are available to visualize the clustering results of selected main lineage cells and the expression of selected genes in the selected main lineage cells (Figure 2C). To further help users explore the functions of each cell type, an interactive heatmap is implemented in a sub-tab to display the pathway enrichment score for each cell type. In the “differential expression analysis” tab, an interactive heatmap in a sub-tab showing the differential expression of genes in each cell type between tumor and normal or between immunotherapy responders and non-responders is presented to allow users to explore anti-tumor immunity and immunotherapy biomarkers (Figure 2D). For detailed information, UMAP plots and barplots are available to visualize the differential expression of the selected gene between different groups in the selected cell type (Figure 2D). Similarly, an interactive heatmap is implemented in a sub-tab to display the difference in pathway enrichment scores between different groups in each cell type. In the “co-expression analysis” tab, users can calculate the correlation between the expression of a gene of interest and that of other genes or calculate the expression of gene pairs in different cell types (Figure 2E). In the “trajectory analysis” tab, users can obtain the expression of genes of interest in the pseudotemporal ordering of cells inferred by Monocle 2 (Figure 2F). The “cell–cell communication” tab allows users to obtain crosstalk between different cell types inferred by receptor–ligand expression (Figure 2G).

#### Immunotherapy response

**Figure 3:**
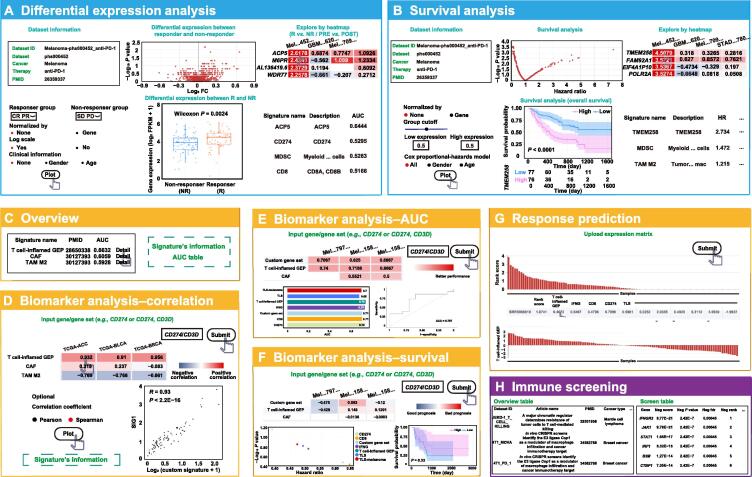
**Web interface and usage of “immunotherapy response”, “response signature”, and “immune screening” modules in TIGER** **A.** Differential expression analysis tab in the “immunotherapy response” module. First in the first row: data source information. Second in the first row: overview of differential expression analysis results. Third in the first row: heatmap showing the results of differential expression analysis between responder and non-responder or between pre-treatment and post-treatment. First in the second row: panel showing the parameter options. Second in the second row: boxplot visualizing the differential expression of the selected gene. Third in the second row: table displaying the performance of the known immunotherapy prediction signature. **B.** Survival analysis tab in the “immunotherapy response” module. First in the first row: data source information. Second in the first row: overview of the survival analysis results. Third in the first row: heatmap displaying the survival analysis results in all datasets. First in the second row: panel showing the parameter options. Second in the second row: KM plot visualizing the survival difference of the selected gene in the selected dataset. Third in the second row: table displaying the performance of the known immunotherapy prediction signature. **C.** Overview tab in the “response signature” module. Left panel showing an overview of known immunotherapy response signature. Right panel showing an overview of AUC table in 40 datasets receiving immunotherapy. **D.** Interface of the “response signature” module for the correlation analysis between the gene of interest and the known gene signature using the TCGA gene expression data. **E.** Interface of the “response signature” module for comparing the performance of user-defined biomarkers with known immunotherapy response signatures using the immunotherapy gene expression data by AUC metric. **F.** Interface of the “response signature” module for comparing the performance of user-defined biomarkers with known immunotherapy response signatures using the immunotherapy gene expression data by HR. KM plot visualizing the survival difference of the selected gene in the selected dataset is shown at the lower-right corner. **G.** Interface of the “response signature” module for predicting the immunotherapy response based on user-provided gene expression data. **H.** Interface of the “immune screening” module for exploring CRISPR datasets related to immunotherapy. KM, Kaplan–Meier; TCGA, The Cancer Genome Atlas; HR, hazard ratio; GEP, gene expression profile; CAF, cancer-associated fibroblast; TLS, tertiary lymphoid structure; TAM M2, tumor-associated macrophage M2; CRISPR, clustered regularly interspaced short palindromic repeats.

This module provides many functions for the analysis of bulk transcriptome gene expression data using clinical immunotherapy information. This module consists of three tabs including “differential expression analysis”, “survival analysis”, and “gene set query”. In the “differential expression analysis” tab, users can browse the data source information and obtain an overview of the differential expression analysis results. Two interactive heatmaps displaying differentially expressed genes between responders and non-responders or between pre- and post-therapy conditions are presented to allow users to explore immunotherapy response biomarkers and resistance mechanisms (Figure 3A). By clicking a cell on the heatmap, users will obtain detailed information on the differential expression of the selected gene between responders and non-responders or between pre- and post-therapy conditions in the selected dataset. For detailed information, a boxplot is presented to visualize the differential expression (Figure 3A). In addition, users can adjust parameters, such as group, gene normalization, data scale, and clinical classification for differential expression analysis (Figure 3A). A table is displayed to allow users to compare the performance of the selected gene with that of the known immunotherapy prediction signature (Figure 3A). In the “survival analysis” tab, users can browse the data source information and obtain an overview of the survival analysis results (Figure 3B). The “survival analysis” tab presents an interactive heatmap displaying the survival analysis results to allow users to evaluate immunotherapy response biomarkers (Figure 3B). Detailed information is presented when users click on a cell in the interactive heatmap. For detailed information, a Kaplan–Meier (KM) plot is provided for visualization (Figure 3B). Moreover, users can adjust the survival analysis parameters and compare their performance with those of known signatures (Figure 3B). To allow users to evaluate their own gene signatures using our collected immunotherapy gene expression datasets, we designed the “gene set query” tab.

#### Response signature

The “response signature” module contains analysis functions for exploring cancer immunotherapy using known immunotherapy response signatures collected from public literature. Users can select a published signature and click on the details to determine the performance of the signature in 23 independent datasets. The area under the curve (AUC) metric was used (Figure 3C). First, users can check whether the genes of interest correlate with the known immunotherapy response signature using TCGA gene expression data without clinical immunotherapy information (Figure 3D). Second, users can compare the performance of their own biomarkers with known immunotherapy response signatures using gene expression data with clinical immunotherapy information (Figure 3E and F). Third, users can predict patient immunotherapy responses by applying published gene signatures to user-provided baseline gene expression profiles (Figure 3G).

#### Immune screening

The “immune screening” module presents the basic information of all immune screens in a table. This table contains “Screen ID”, “Article name”, “PMID”, “Cancer type”, “Dataset type”, “Cell line”, “Species”, “Condition”, “Analysis”, and “Size”. Users can view the specific content on the screen by selecting a screen ID. After selection, the article source of the data and specific information of the article are displayed to the user. Because the screen data are mainly analyzed using two different pipelines, different display schemes for the data from different analysis sources have been provided (Figure 3H).

#### Quick search

Users can quickly obtain comprehensive analytical results of the aforementioned four modules by searching for a gene of interest.

### Integrative analysis using TIGER reveals an effective predictor of immunotherapy response

We next performed a systematic analysis using TIGER to show its value in facilitating research related to cancer immunotherapy. Current immunotherapy studies have mainly focused on CD8^+^ T cells to explore the mechanisms of immunotherapy-induced anti-tumor immunity and discover effective immunotherapy response biomarkers. Among the tumor-infiltrating T lymphocytes (TILs), apart from CD8^+^ T cells, it is also known that CD4^+^ T cells play important roles in anti-tumor immunity, *e.g.*, the activation and growth of cytotoxic CD8^+^ T cells. However, the role of CD4^+^ T cell response to immunotherapy in TME has seldom been studied. Here, we integrated bulk and single-cell transcriptome gene expression data in TIGER to comprehensively explore the anti-tumor immunity of CD4^+^ T cells under immunotherapy.

**Figure 4:**
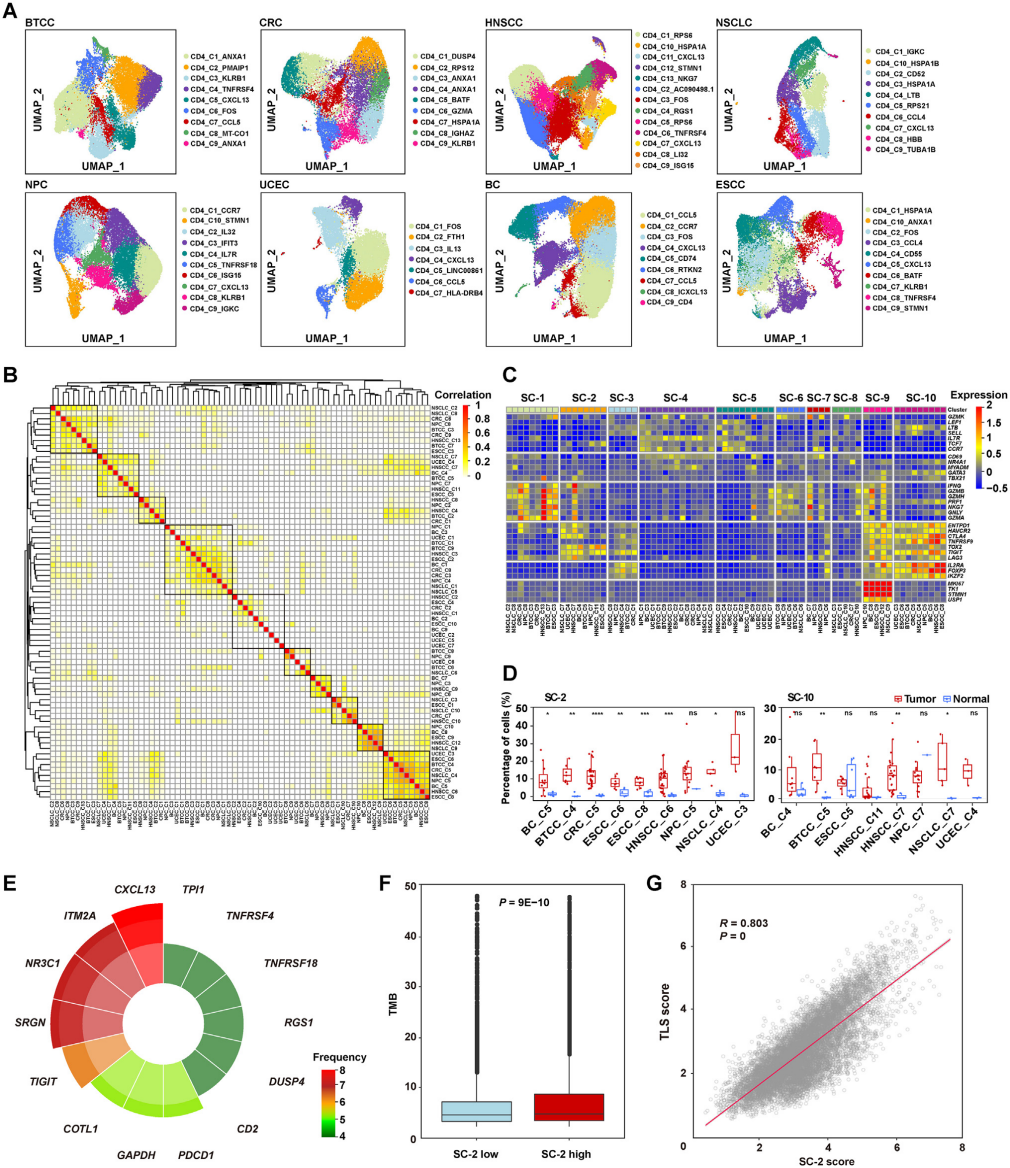
**Tumor-enriched CD4^+^ T cells revealed by pan-cancer analysis of TIGER resource** **A.** UMAP plots showing the cell types of CD4^+^ T cells derived from the clustering of scRNA-seq data of eight cancer types. **B.** Heatmap showing the hierarchical clustering of all the cell types of CD4^+^ T cells of the eight cancer types. The 10 super cell types are highlighted in black boxes. **C.** Heatmap showing the expression of classical immune cell markers for naïve, memory, effector, exhausted, Treg, and proliferating cells in each super cell type. **D.** Boxplots showing the two CD4^+^ cell types (SC-10 and SC-2) that are universally found in all cancer types and consistently enriched in tumor samples. *, *P* < 0.05; **, *P* < 0.01; ***, *P* < 0.001; ****, *P* < 0.0001; ns, not significant (*t*-test). **E.** Genes that are significantly up-regulated in the SC-2 CD4^+^ T cells in at least four cancer types (FC > 1.5, *P* < 0.05). **F.** The difference in TMB between TCGA tumor samples with low and high SC-2 gene signature scores. **G.** The correlation between TLS gene signature (*CCL9*, *CCL21*, *CXCL13*, *CCR7*, *SELL*, *LAMP3*, *CXCL4*, *CD86*, and *BCL6*) and SC-2 signature. BTCC, bladder transitional cell carcinoma; CRC, colorectal cancer; HNSCC, head-neck squamous cell carcinoma; NSCLC, non-small-cell lung carcinoma; NPC, nasopharyngeal carcinoma; UCEC, uterine corpus endometrial carcinoma; BC, breast cancer; ESCC, esophageal squamous cell carcinoma; TMB, tumor mutational burden; TLS, tertiary lymphoid structure.

To this end, we selected cancer types with at least 10,000 CD4^+^ T cells for pan-cancer analyses. As a result, 176,371 CD4^+^ T cells from 8 cancer types were used for downstream analysis. We could determine 79 cell types by separately clustering the CD4^+^ T cells in each cancer type, ranging from 7 to 12 cell types in each cancer type (Figure 4A). Unsupervised clustering of the 79 CD4^+^ T cell types revealed 10 super cell types across different cancer types (Figure 4B). Differential expression analysis revealed that SC-1 cells are effector cells, as they highly express effector markers (*GZMA*, *IFNG*, and *GNLY*); SC-4 cells are naïve cells, as they highly express naïve markers (*TCF7* and *CCR7*); SC-9 cells are proliferating cells, as they highly express proliferating markers (*MKI67* and *STMN1*); and SC-10 cells are Treg cells, as they highly express Treg markers (*FOXP3* and *IL2RA*) (Figure 4C). SC-2, SC-9, and SC-10 cells were exhausted, as indicated by the high expression of exhaustion markers (*TOX2* and *TIGIT*) (Figure 4C). Interestingly, in addition to SC-10 cells (Treg cells), SC-2 cells were universally found in all cancer types and were consistently enriched in tumor samples (Figure 4D). SC-2 cells are enriched and exhausted in tumors, suggesting that these cells might play a regulatory role in anti-tumor immunity. Then, a pan-cancer differential expression analysis was performed, and we found that genes such as *CXCL13*, *ITM2A*, *NR3C1*, *SRGN*, *COTL1*, and *PDCD1* were significantly up-regulated in SC-2 cells compared with other cells in at least five cancer types, but not in SC-10 cells (Figure 4E). These genes were used as gene signatures to represent the SC-2 cells. By applying this SC-2 gene signature to TCGA dataset, we found that patients with higher SC-2 gene signature scores had a significantly higher tumor mutation burden (Figure 4F), further indicating the regulatory role of SC-2 cells in anti-tumor immunity. Interestingly, we also observed the expression of effector markers, such as *IFNG* in SC-2 cells (Figure 4C), indicating that SC-2 cells might function as cytotoxic T cells to directly kill tumor cells, as reported in a previous study [8]. Moreover, *CXCL13* is highly expressed in the tertiary lymphoid structure (TLS) and plays a central role in its formation. TLS has been found to modulate anti-tumor immune activity and is associated with immunotherapy responses [34], [35], [36]. We hypothesized that SC-2 CD4^+^ cells with high *CXCL13* expression might also regulate anti-tumor immunity by assisting TLS formation. Indeed, we found that the SC-2 gene signature was strongly positively correlated with the TLS gene signature in TCGA pan-cancer datasets (Pearson’s correlation = 0.803) (Figure 4G).

**Figure 5:**
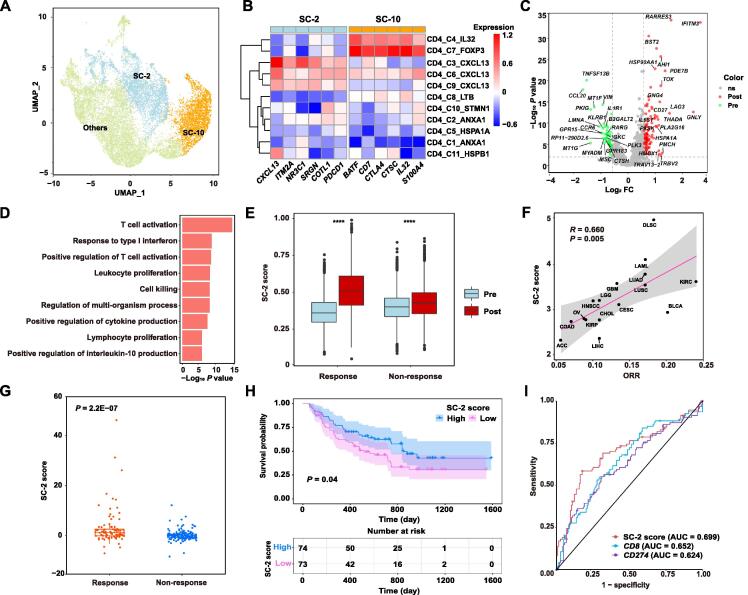
**A gene signature of a subset of tumor-enriched CD4^+^ T cells predicts immunotherapy response** **A.** UMAP plot showing the clustering results of CD4^+^ T cells of BCC. **B.** The expression of marker genes for SC-2 and SC-10 in the subsets of CD4^+^ T cells of BCC. **C.** Differentially expressed genes between SC-2 cells of post-therapy and pre-therapy responder samples. **D.** Significantly enriched pathways in post-therapy SC-2 cells. **E.** Differential expression of the up-regulated genes upon therapy in responders between pre-therapy and post-therapy non-responder samples. ****, *P* < 0.0001 (*t*-test). **F.** The correlation between immunotherapy response ORR and SC-2 score. **G.** The differential SC-2 gene signature score between immunotherapy (anti-PD-1 or anti-CTLA4) responders and non-responders. **H.** Survival difference between patients with high and low SC-2 scores. **I.** Response prediction performance comparison among SC-2 score, *CD8* (average of *CD8A* and *CD8B*) expression, and *PD-L1* (*CD274*) expression. BCC, basal cell carcinoma; ORR, objective response rate.

Next, we explored whether SC-2 CD4^+^ T cells played a role in response to immunotherapy. By analyzing 16,194 CD4^+^ cells from scRNA-seq data of basal cell carcinoma (BCC) and immunotherapy response data, we revealed that the SC-2 CD4^+^ cells existed in the TME of BCC (Figure 5A and B). Differential expression analysis between pre- and post-therapy conditions in SC-2 CD4^+^ T cells of immunotherapy responders revealed that effector genes such as *GNLY* and *IFITM3* were significantly up-regulated in post-therapy cells (Figure 5C). Moreover, immune activation pathways such as T cell activation and response to type I interferon were obviously enriched in SC-2 CD4^+^ T cells after immunotherapy (FC = 1.38) (Figure 5D). However, these genes were only slightly up-regulated in the SC-2 CD4^+^ T cells of non-responders after immunotherapy (FC = 1.06) (Figure 5E). These results suggest that SC-2 CD4^+^ T cells may play an important role in response to immunotherapy. By applying the gene signature of SC-2 CD4^+^ T cells to TCGA dataset, we found that the average gene signature score of cancer types was significantly associated with the objective response rate (ORR) of the corresponding cancer types (Pearson correlation = 0.66) (Figure 5F). We then applied the SC-2 gene signature to five melanoma immunotherapy datasets, including 263 samples with anti-PD1 or anti-CTLA4 therapies, and found that a higher gene signature score was not only significantly associated with better responses (Figure 5G), but was also significantly associated with better survival under immunotherapy (Figure 5H). This gene signature was superior to that of other known biomarkers, such as *CD8* and *PD-L1* (Figure 5I). Taken together, we discovered a subset of CD4^+^ T cells that can modulate anti-tumor immunity and predict immunotherapy responses by the integrative analysis of the resources in TIGER.

## Discussion

TIGER is an interactive web-accessible portal for the integrative analysis of bulk and single-cell transcriptomic gene expression data related to cancer immunotherapy.

Compared with other existing tools, such as TCIA, TIDE, and TISCH, TIGER has several advantages. First, TIGER is the first web server to integrate bulk and single-cell gene expression data to discover anti-tumor immunity mechanisms and response biomarkers in cancer immunotherapy. Second, TIGER holds the most comprehensive transcriptomic gene expression data related to cancer immunotherapy, with non-immunotherapy bulk gene expression data for 11,057 tumor/normal samples across 33 cancer types, immunotherapy bulk gene expression data for 1508 tumor samples across 8 cancer types, and single-cell gene expression data for 2,116,945 cells of 655 samples across 25 cancer types. Third, TIGER contains more analysis and visualization functions for both bulk and single-cell gene expression analyses than the other tools. In particular, differential analysis between tumor and normal cells and between different cell types using scRNA-seq data allows users to explore anti-tumor immunity and develop gene signatures of specific cell types. The analysis of immunotherapy gene expression data, together with public gene signatures, allows users to comprehensively evaluate biomarkers of immunotherapy responses.

In conclusion, the analysis of bulk and single-cell data in the same platform specialized for cancer immunotherapy research will facilitate users to gain more insights into cancer immunotherapy. In the future, we will continually update the TIGER database by integrating new bulk and single-cell gene expression data. We plan to add T cell receptor (TCR) and B cell receptor (BCR) sequencing data to TIGER to further facilitate the understanding of tumor immunology. Continuous efforts will be made to implement new analytical and visualization functions to improve the performance of TIGER.

## Data availability

TIGER is freely accessible at http://tiger.canceromics.org/.

## Competing interests

The authors have declared no competing interests.

## CRediT authorship contribution statement

**Zhihang Chen:** Formal analysis, Investigation, Data curation. **Ziwei Luo:** Investigation, Visualization. **Di Zhang:** Data curation. **Huiqin Li:** Software. **Xuefei Liu:** Formal analysis, Investigation, Data curation, Writing – original draft. **Kaiyu Zhu:** Software, Formal analysis, Investigation. **Hongwan Zhang:** Data curation. **Zongping Wang:** Data curation. **Penghui Zhou:** Conceptualization, Writing – review & editing. **Jian Ren:** Conceptualization, Writing – review & editing. **An Zhao:** Conceptualization, Writing – review & editing. **Zhixiang Zuo:** Conceptualization, Supervision, Writing – review & editing. All authors have read and approved the final manuscript.

## Supplementary material

Video S1 **Instructional video for TIGER** This video introduces the usage of TIGER in detail. TIGER, tumor immunotherapy gene expression resource.
